# Single Nanoparticle Plasmonic Sensors

**DOI:** 10.3390/s151025774

**Published:** 2015-10-12

**Authors:** Manish Sriram, Kelly Zong, S. R. C. Vivekchand, J. Justin Gooding

**Affiliations:** 1 School of Chemistry, The University of New South Wales, Sydney 2052, Australia; E-Mails: m.sriram@unsw.edu.au (M.S.); k.zong@unsw.edu.au (K.Z.); 2 Australian Centre for NanoMedicine, The University of New South Wales, Sydney 2052, Australia; 3 ARC Centre of Excellence in Bio-Nano Science & Technology, The University of New South Wales, Sydney 2052, Australia

**Keywords:** surface plasmons, sensors, metal nanoparticles, single molecule detection, optical sensors

## Abstract

The adoption of plasmonic nanomaterials in optical sensors, coupled with the advances in detection techniques, has opened the way for biosensing with single plasmonic particles. Single nanoparticle sensors offer the potential to analyse biochemical interactions at a single-molecule level, thereby allowing us to capture even more information than ensemble measurements. We introduce the concepts behind single nanoparticle sensing and how the localised surface plasmon resonances of these nanoparticles are dependent upon their materials, shape and size. Then we outline the different synthetic approaches, like citrate reduction, seed-mediated and seedless growth, that enable the synthesis of gold and silver nanospheres, nanorods, nanostars, nanoprisms and other nanostructures with tunable sizes. Further, we go into the aspects related to purification and functionalisation of nanoparticles, prior to the fabrication of sensing surfaces. Finally, the recent developments in single nanoparticle detection, spectroscopy and sensing applications are discussed.

## 1. Introduction

The interaction of light with matter is one of the fundamental ways to characterise and understand the properties of matter. When light interacts with certain metals (e.g., Au, Ag), it sets off a collective oscillation in conduction band electrons at the metal-dielectric interface [1]. These collective oscillations, termed as surface plasmons, provide an elegant way to couple and confine electromagnetic radiation in sub-diffraction volumes. The dielectric properties of the metal and the surrounding material play an important role in determining the optical properties of the plasmonic structures. In the case of nanostructured plasmonic metals, such as metallic nanoparticles, the frequency and strength of these resonances depend on the size and shape of the metallic nanostructure. The intense electromagnetic fields associated with plasmonic nanostructures have been exploited to enhance different optical processes such as fluorescence [2] and Raman scattering [3]. In addition, plasmonic nanoparticles have been applied for the fabrication of nanoscale lasers [4], targeted cancer therapy [5], as non-blinking imaging markers [6] and nanoscale sensors [7].

Nanoscale sensors for biological applications has become an important application for plasmonic nanoparticles. With an increasing drive towards personalised medicine, the development of the next-generation of biosensors, capable of detecting single binding events with individual nanostructures, is rapidly becoming a reality. Single molecules have been detected by a wide variety of method, which include nanoscale transistors made from carbon nanotubes [8,9,10], whispering gallery modes [11,12,13], optical traps [14,15] and a wide range of fluorescence-based methods [16].

Single nanoparticle sensors with single molecule sensitivity would offer the unique advantage of resolving rare events and the distribution of molecular properties; information that would otherwise be lost in conventional ensemble methods [17,18,19]. Utilizing individual nanoparticles as independent transducers also offers the potential of low detection limits, high-throughput assays through multiplexing and require smaller sample volumes. However, it is important to consider the effects of mass transport, as it plays an important role in determining the sensitivity and response time of single nanoparticle based sensors [20]. Another advantage shared with plasmonic biosensors is the capability for miniaturization and eventual development into point-of-care devices [21].

In this review, we primarily focus on the different approaches for the construction of single particle plasmonic sensors, with an emphasis on pushing the detection limit towards single molecules. There are many techniques being developed with the aim of achieving single-molecule detection, such as fluorescence-based methods. However this work only reviews the advances in plasmonic-based single particle sensors. The review is divided into four sections; in the first section, we briefly review the synthesis of plasmonic nanoparticles, followed by the functionalisation and surface modification of these nanoparticles. We then discuss the different parameters involved in the design of surface plasmon sensors. In the fifth section, we discuss the prominent examples in the literature on single nanoparticle sensors and the optical techniques employed. Finally, we conclude the review by outlining the challenges limiting the single plasmonic sensors and possible future directions.

## 2. Synthesis/Fabrication of Plasmonic Nanoparticles

**Figure 1:**
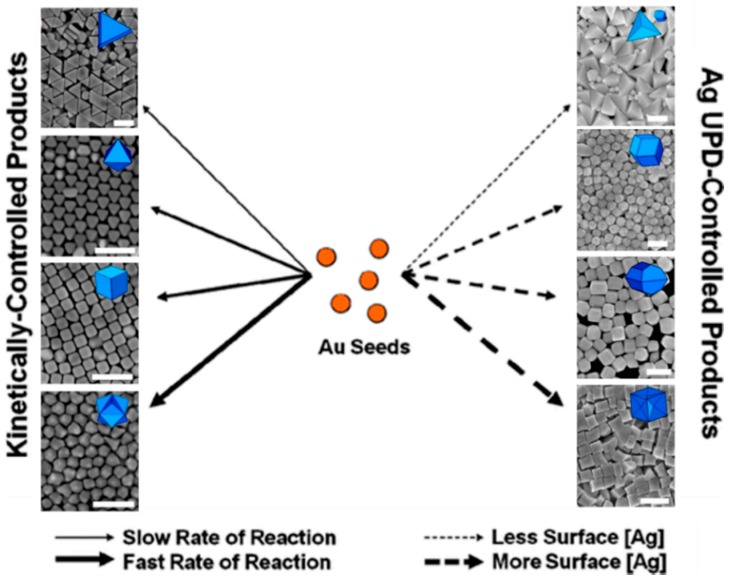
General rules of shape-control in silver-assisted, seed-mediated gold nanoparticles synthesis, where the shape of nanoparticles can be controlled simply by altering the reaction rate or altering the silver concentration. Scale bars are 200 nm. Adapted with permission from reference [12]. Copyright 2012 American Chemical Society.

In this section, we review the recent advances in the preparation of plasmonic nanostructures for sensing. Two prominent approaches have been employed in the literature: (1) bottom-up chemical procedures that allow for the large-scale synthesis of plasmonic nanostructures with a wide range of shapes and sizes (see Figure 1). After synthesis, these nanostructures are then modified with suitable functional molecules that allow for bio-recognition along with an anti-fouling surface. These nanostructures can be assembled on a surface to yield plasmonic chips [22] or dispersed into cells to probe intracellular concentration of important biomarkers [23]; (2) Top-down approaches such as lithography combined with metal-deposition allow for the precise fabrication of plasmonic nanostructures. The optical properties of the targeted structures are first optimised with computational electromagnetic simulations. This top-down approach has also allowed for the careful elucidation of the role played by different structural parameters on the sensing performance of the nanostructures. In this review, the synthetic approaches for top-down methods are not discussed in detail, however an excellent review by Estevez *et al.* [24] discusses the aspects of top-down sensor fabrication.

Although, the chemical synthesis strategy yields large sample volumes, these methods used to be plagued by polydispersity and challenges associated with surface modification. However, the emergence of well-defined strategies for the synthesis of plasmonic nanocrystals with desired size and shape characteristics, have made them attractive for surface plasmon based sensing applications. One of the simplest means of producing large quantities of Au nanospheres remains the citrate reduction of HAuCl_4_, as reported by Turkevich in 1951 [25]. The Turkevich method has been optimised further to yield Au nanospheres with controlled sizes by modifying the pH of the reaction solution [26] or the concentration of chloride ions [27].

**Figure 2:**
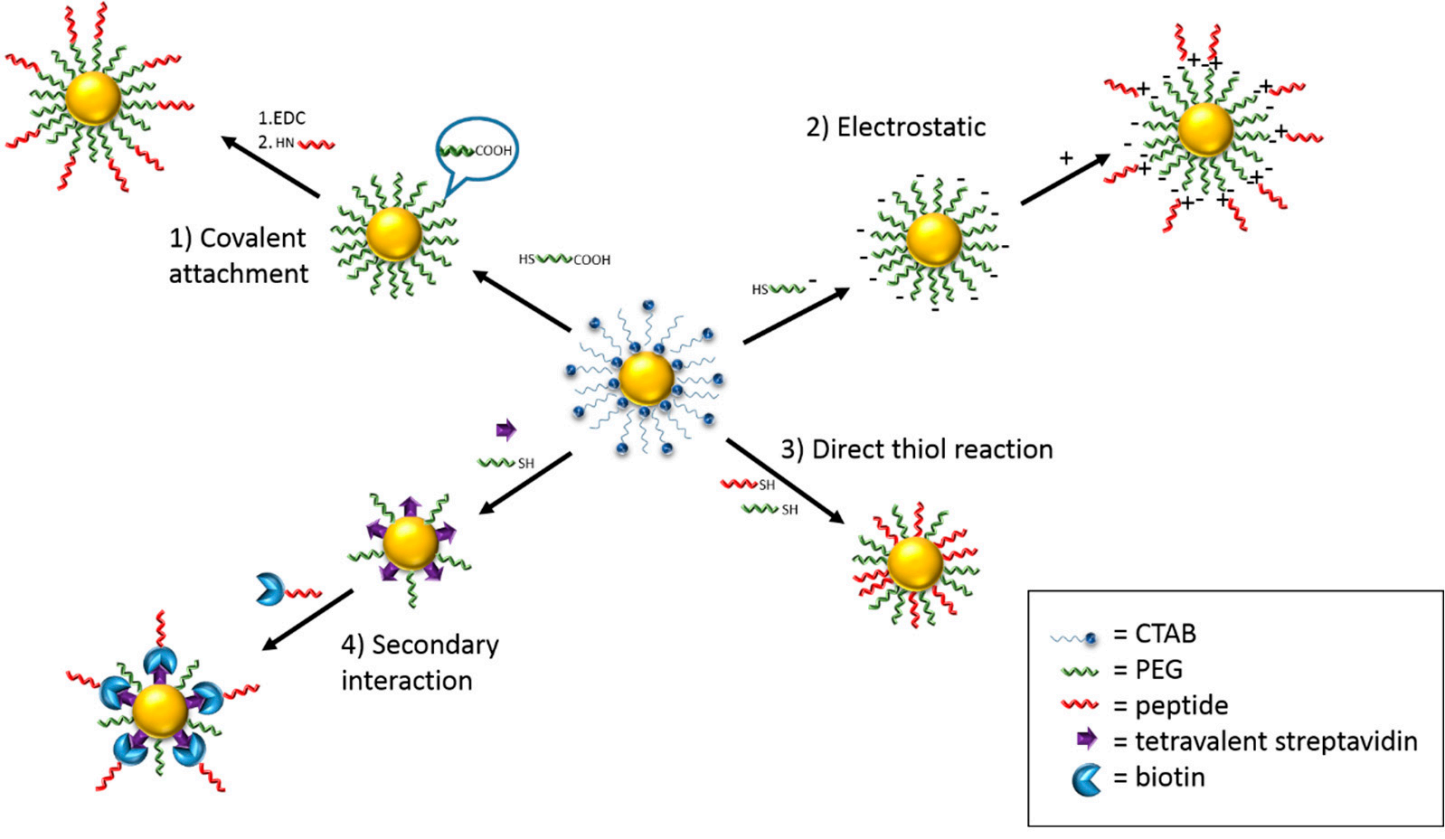
The four major surface modification approaches for functionalisation of nanoparticles, given a peptide as an example of a biomolecule and PEG as the antifouling material. (**1**) Covalent attachment: covalent reactions such as EDC/NHS coupling occur between the antifouling layer and biomolecule end groups; (**2**) Electrostatic: use cationic/anionic antifouling materials and biomolecules to allow charge-charge conjugation; (**3**) Direct thiol reaction: both anti-fouling material and biomolecules loaded through thiol binding to the surface; (**4**) Secondary interaction: ligands are loaded onto the surface followed by the biomolecules containing a specific receptor to allow ligand-receptor specific conjugation.

The advances in the shape-controlled synthesis of Au nanocrystals can be attributed to the exploitation of surfactants such as hexadecyltrimethylammonium bromide (CTAB) and the slow overgrowth in the presence of weak reducing agents, such as ascorbic acid. Jana *et al.* [28], reported the first paper on the seed-mediated growth of Au nanorods. In this method, small CTAB-capped Au nanoparticles (3.5 nm in diameter), termed as “seeds” are first prepared by the borohydride reduction of HAuCl_4_. In the second step, the seeds are introduced into a growth medium consisting of ascorbic acid, CTAB and HAuCl_4_. The ascorbic acid slowly reduces Au^3+^ to Au^0^, which then deposits on the seeds, causing them to grow. The CTAB bilayer surrounding the Au nanospheres aids in anisotropic growth. Nikoobakht and El-Sayed [29] demonstrated that the length of the Au nanorods prepared by the seed-mediated method can be controlled by the addition of silver ions to the growth solution. Subsequently, the seed-mediated technique has been employed for the preparation of Au nanospheres [30], nanocubes [31], nanobipyramids, nanostars [32] and other nanostructures [33]. The size and shape of the nanoparticles can be controlled by adjusting certain parameters during synthesis. These parameters include concentration of reagents [34], inclusion of halides [35], type and concentration of reducing agents used [36], reaction temperature [37,38] and pH [39]. Furthermore, the seed-mediated protocol has been further modified into a one-step “seedless” method, wherein the seeds are generated *in situ* with sodium borohydride (see Figure 2) [40]. This modification allows for the large-scale production of Au nanospheres, nanoplates, and nanostars. Recently, Xu and co-workers [36] have synthesised Au nanorods with large aspect ratios (up to 20.6), using the seedless method, by adding hydrochloric acid and using hydroquinone as the reducing agent.

The size and shape distribution of plasmonic nanoparticles also plays a crucial role in the construction of plasmonic sensors. Monodispersity can be obtained with two different approaches: by fine-tuning experimental parameters during synthesis or with size/shape based separation after synthesis. While the synthetic approach for different nanoparticle shapes have developed over the years, there has been an increased interest in optimizing for better monodispersity. Wu *et al.* [41] have reported a protocol for the preparation of Au nanorods with good monodispersity as well as high aspect ratios. The monodispersity of the nanorods was improved by the addition of nitric acid to the growth solution. The authors conclude that the nitrate ion rather than the pH change, promotes formation of a uniform one-dimensional structure. A recent synthetic approach reported by Ye and co-workers [42], where an aromatic additive aids in improving the monodispersity. Aromatic additives such as sodium salicylate are believed to stabilise the interface between Au and the CTAB bilayer, thereby yielding highly monodispersed gold nanorods.

Nanoparticles with specific sizes and shapes can be obtained from polydispersed samples with the help of size and shape based separation techniques. Simple fractionated centrifugation of an aqueous suspension results in the large and unstable aggregations being separated from the rest of the suspension. In a similar manner, a fractionated precipitation technique is reported by Thai *et al.* [43] which involved the addition of sodium chloride or ethanol to change solubility of PEG-modified nanoparticles. Other separation techniques such as filtration [44], chromatography [45] and force-induced separation [46] could also provide size- and shape-controlled separation. Post-synthesis separation techniques could be used in combination with a synthetic approach, to provide highly monodisperse, shape- and size-controlled nanocrystals for the preparation of single particle plasmonic sensors.

Density gradient centrifugation is a general and non-destructive separation technique, which may be used in the post-synthesis purification of nanocrystals. The separation is reliant on a density gradient, where a centrifugal force is applied to move nanocrystals in a fluid away from the axis of rotation at different speeds according to chemical, structural and dimensional differences of the nanocrystal. The technique could be divided into two types: isopycnic-zonal centrifugation and rate-zonal separation [47]. In isopycnic-zonal centrifugation, a media with a density gradient is used as a liquid column. During prolonged centrifugation, particles move against the density gradient until they stop when reaching the location of the same density, hence fractionated according to their density. However, such a method is limited by the density of aqueous media, and is not suitable for metal nanocrystals due to the high density of metals*.* Rate-zonal separation takes advantage of the difference in sedimentation rate of the particle within the period of centrifugation. Depending on the period of centrifuge and nature of solvent, nanocrystals could be fractionated into different positions. Rate-zonal centrifugation has been reported to be able to sufficiently fractionate metallic nanoparticles in aqueous phase [48,49] as well as in organic phase [50]. More recently, Akbulut and co-workers [51] reported a multi-phase aqueous system, which allows for improved separation of nanoparticles according to their shapes. Rate-zonal centrifugation has an increasing importance in the purification of metallic nanoparticles and is typically used in conjunction with controlling the different growth parameters, in order to produce highly monodispersed metallic nanoparticles.

Centrifugation and separation techniques also allow for the purification of plasmonic nanocrystals from surfactants and starting materials, which often hinder their application, especially when used with cells. The use of surfactants such as CTAB results in well-defined nanoparticles. However, CTAB needs to be replaced with a suitable monolayer that will allow for bio-conjugation and anti-fouling properties [32]. To improve biocompatibility as well as from the perspective of green chemistry, synthetic approaches have been developed using less hazardous substances, involving the replacement of toxic stabilizing and reducing agents with non-toxic plant extracts [52,53]. Such modifications can produce non-toxic nanoparticles (CTAB-free), however with numerous simple purification techniques available, the use of green-chemistry may be more complicated and time-consuming. Hence, for the fabrication of single plasmonic nanoparticle sensor, the well-established seed-mediated or seedless surfactant-assisted approach is preferred.

## 3. Functionalisation of Metallic Nanoparticles

The synthesis of nanoparticles results in certain capping groups, it is generally required to further modify them to prepare them for application. Such modifications include the removal of toxic surfactants for improved biocompatibility, functionalising the surface with ligands for specific sensing purposes and enhanced signals, and separation techniques for improving size and shape distribution. Place exchange of the capping group with the required ligand is one of the most commonly used methods. As gold is the major material used in single nanoparticle sensing devices, alkanethiols have become the surface chemistry of choice, due to the high affinity of thiols to gold. The most widely used compounds are thiol-terminated polyethylene glycol (HS-PEG) [54,55,56,57,58], due to their high biocompatibility, resistance to non-specific protein adsorption (anti-fouling property) and ability to stabilise the shape of the nanoparticles in multiple mediates [54,55,56,57,58,59]. The standard PEGylation method was reported by Liao and Hafner in 2005, simply with the addition of HS-PEG at low CTAB concentration and an overnight incubation [54]. This approach was further adapted by Liu *et al.* [60], which involves the use of Tween-20 to stabilise the gold nanorods, bis(*p*-sulfonatophenyl)phenylphosphine (BSPP) to activate the surface of the nanorods and sodium chlorides to etch the silver from seed-mediated growth. This approach improved the degree of PEGylation and allows for the complete removal of toxic CTAB, opening the way towards non-toxic, highly biocompatible nanoparticle sensors. In a similar manner, the Au nanosphere sensor reported by Ma *et al.* [61] is modified with thiol-terminated oligoethylene glycol (HS-OEG), using tris-buffer and sodium chloride to promote surface exchange with the thiol. Alkanethiols are also used to exchange CTAB, where modified nanoparticles are dispersible in organic phase [62,63]. Other surface materials such as thiolated CTAB analogues [64], thiolated glycans [65] and phospholipids [57,66] are also reported to successfully replace CTAB and reduce toxicity.

In order to provide selectivity to specific analytes, nanoparticles need to be functionalised with the appropriate biomolecules, such as enzymes [67,68], proteins [69], DNA [61], antibodies [70] or other molecules that provide a suitable sensing interface. This functionalization can be carried out along with the removal of the surfactant during the place exchange reaction described above, by using thiol-containing biomolecules. The plasmonic sensor reported by Ma *et al.* [61] which was discussed earlier, used thiolated-DNA in a mixture with HS-OEG, where the thiolated-DNA provides binding affinity for the analyte and HS-OEG provides complete coverage of the nanoparticle surface. In a study by Zijlstra and co-workers [67], thiolated biotin was used for both exchanging CTAB and functionalization. An alternative strategy is to use compounds with a carboxyl or primary amine end group as an alternative to CTAB, followed by a conjugation reaction to further functionalise the nanoparticle. Common conjugation reactions include charge-charge conjugation, ligand-receptor conjugation, or covalent attachment. An example of covalent attachment is EDC/NHS or EDC/sulfo-NHS conjugation, where 1-ethyl-3-(3-dimethylaminopropyl)-carbodiimide (EDC) is used as a crosslinking agent, *N*-hydroxysuccinimide (NHS) [71] or *N*-hydroxysulfosuccinimide (sulfo-NHS) as a stabilising agent for the active ester intermediate [72,73,74]. This results in the formation of an amide bond between the primary amine and a carboxyl or phosphate group. EDC/NHS or EDC/sulfo-NHS allows a large range of molecules to be loaded onto the nanoparticle surface [68,69,70,71,72,73], which opens possibilities for highly specific and multifunctional sensors.

Another modification strategy involves the use of plasmonic nature of nanoparticles, where conjugation is driven by an optical pulse at certain wavelengths. For example, Bisker *et al.* [75] reported the laser-controlled conjugation of a range of fluorescent proteins to PEG-modified gold nanoparticles, enabling the detection of protein adsorption to gold nanoparticles. Furthermore, site-specific modification is also utilised in order to selectively modify certain sides of a nanostructure, such as the tips of gold nanorods [76] or edges/vertices of gold triangles [77]. Such modifications allow for more specific conjugation of the gold nanoparticles, which can improve LSPR responses as these sharp points are generally more sensitive to refractive index changes [78].

## 4. Design of Surface Plasmon Sensors

Au is the primary material choice for the construction of individual plasmon sensors due to the excellent chemical stability of Au. Although silver displays better surface plasmon characteristics than gold, it is easily prone to oxidation and therefore limiting its application for sensing [79]. The individual surface plasmon sensors that are discussed in this review are optical sensors that display a change in the surface plasmon resonance upon the binding of a molecule or plasmonic nanoparticles or enzyme-linked immunoprecipitation. These sensors exploit the fact that localised surface plasmons are sensitive to the refractive index of the surrounding medium and are nanoscale analogues of prism-based SPR methods that have been commercially employed for decades. The size, shape and the material properties play a crucial role in determining the sensitivity of the nanoparticle to refractive index changes. Chen *et al.* [80] have looked into the effect of the nanoparticle size and shape on bulk refractive index sensitivity for gold nanoparticles. They observed that the bulk refractive index sensitivity of anisotropic nanoparticles (for e.g., nanobranches and nanorods) was significantly higher than that of nanospheres. However, as the sensitivity of the plasmonic system is pushed towards single molecules, it is crucial to understand the local effects on the refractive index as compared to bulk.

Zhang and Martin [81] have worked out the relationship between the changes in the surface plasmon resonance upon the trapping/adsorption of a single nanoparticle. They numerically show that the resonance shift is linearly related to the product of the local electric field intensity of the resonance mode, the material dispersion factor and polarizability of the nanoparticle.

It is well known that the electromagnetic fields associated with surface plasmons decay exponentially and the capture molecules occupy a significant part of electromagnetic field region. As the molecule of interest only affects the refractive index of a particular layer, it crucial to understand the effects on local refractive index changes as compared to bulk. Recently, Li *et al.* [82], have looked at the surface sensing performance of diffractively coupled plasmonic systems. In their experiments, they controlled the refractive index surrounding a periodic nanodisc array with the atomic layer deposition of Al_2_O_3_. Based on their experiments, they conclude that the surface sensing capacity cannot be described by bulk refractive index sensitivity and one has to take into consideration the decay of the electromagnetic fields.

## 5. Towards Single Molecule Sensing with Individual Nanoparticles

Single particle sensing approaches utilise the LSPR of nanoparticles with the sensitivity of the LSPR signal for sensing being influenced by the composition, shape, size and local dielectric environment of the nanoparticle. Changes in the refractive index of these nanoparticles induce a shift in the LSPR, which have been observed a dark-field optical microscope and detected with a couple spectrometer or spectrograph [83]. As discussed earlier, numerous shapes, sizes and types of nanoparticles can be used for LSPR and have been investigated for their use in the development of these biosensors [84]. Typically, gold and silver nanoparticles are considered optimal for these detection methods, however silver nanoparticles tend to be passed over in favour of gold nanoparticles due to their increased stability, ease of modification and non-toxic nature [85,86]. For the application of biosensors, Au nanospheres are most commonly used, due to their relative ease of synthesis and modification. However, Au nanorods have also been used for single particle sensing and can offer an improved sensing performance due to the large tunability of aspect ratio, hence the LSPR, and their sharp ends, which increases their sensitivity [24,87,88].

One of the first techniques developed to measure the spectra of single particles utilised total internal reflection microscopy with an imaging spectrometer and was able to measure the spectra of up to 7 particles simultaneously [89]. Another method that was employed to study the spectra of single nanoparticles was differential interference light contrast microscopy, which allowed for the spectra of single silver nanoparticles to be obtained in a time-resolved manner [90]. Dark-field optical microscopy techniques were also studied for their potential application in single particle studies. Using this technique, coupled with a spectrometer, the spectra of single particles were able to be resolved [91,92]. It is important to note that these techniques were limited by the time it took to obtain single-particle spectra and the number of particles that were able to be probed, as the particles had to be focused within the grating of the spectrometer. This also means that the stage had to be moved to obtain spectra of different particles. However, these studies paved the way for single particles to be utilised as sensitive real-time sensing tools [93,94].

In recent years, dark field optical microscopy techniques have been taken further to demonstrate their application as molecular rulers and biosensors. Using this setup, the directed coupling of gold and silver dimers was followed in real time and allowed the study of single DNA hybridisation events [95]. Raschke *et al.* [96] also utilised this technique to detect biotin-streptavidin binding to a concentration of 1 µM on single gold nanorods. Similarly, Sannomiya *et al.* [97] used this technique to demonstrate the detection and quantification of single particle binding events on individual nanoparticles. Being able to monitor these single particle-binding events is a great step forward in the development of single particle biosensors, however it was seen that LSPR shifts were also strongly reliant upon the inter-particle distance, particle position and size. This presents the particular challenges of inconsistent LSPR shifts from different particles, as individual particles are unlikely to be identical in shape, size or mode of binding [98,99].

**Figure 3:**
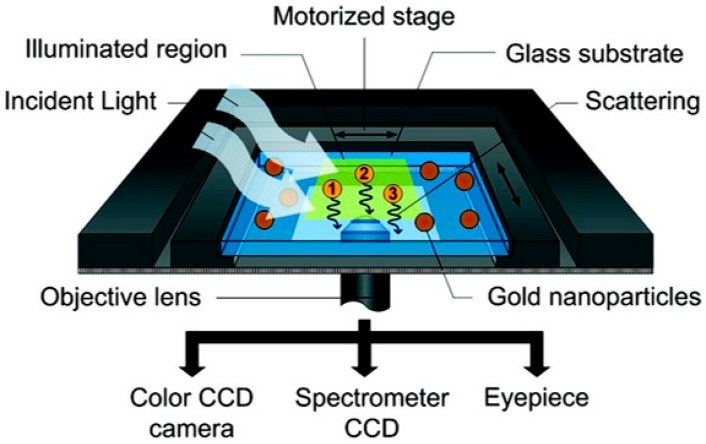
Schematic diagram of the optical setup for the detection of the gold nanoparticles in a nanoarray, where the scattered light is directed to a colour CCD, spectrometer CCD and the eyepiece. The nanoarray is spectroscopically analysed by scanning along the region-of-interest in sequential steps. The single-particle spectral information is then recreated into a pseudo-image for further analysis. Adapted with permission from [101]. Copyright 2011 American Chemical Society.

Another technical limitation of single particle spectroscopy methods is obtaining spectral information from a large group of nanoparticles. These particular hurdles can be overcome by coupling the dark-field microscope with an imaging spectrograph, capable of obtaining single particle spectra of multiple nanoparticles simultaneously [24]. A conventional spectrometer requires the nanoparticles to be focused within the small entrance slit. However, by using an imaging spectrometer spectral information is obtained from a larger field of view. In a recent study by Nusz *et al.* [100], a line-spectrometer was used to detect streptavidin binding on single gold nanorods. Using this technique, more nanoparticles were able to be probed more rapidly, whilst also demonstrating a significant increase in sensitivity. In an effort to increase the amount of nanoparticles being probed, a nano-array based sensor, made of 30 DNA-modified Au nanorods, was developed and used to identify and monitor spectral shifts in response to thrombin binding (see Figure 3) [101]. A novel normalization technique was also developed, based on individual sensitivity to refractive index change, allowing all nanorods (and differently shaped nanoparticles as reported) to be probed simultaneously and showing shifts, which can be correlated to those of the other particles [101]. This work addressed two key issues facing single nanoplasmonic sensors, being able to probe numerous nanoparticles in a two-dimensional array, permitting the analysis of multiple particles, and the inconsistent LSPR shifts due to particle-particle variations. However, it is noted that this method requires relatively long acquisition times due to step-wise spectral scanning.

One of the approaches to speed up single nanoparticle spectroscopy is the use of liquid crystal tunable filters coupled with CCD detectors [102]. This approach enables hyperspectral imaging of single nanoparticles. Kall and co-workers [103] demonstrated this procedure with the enzymatic catalysed precipitation of 3-diaminobenzene (DAB) onto lithographically-fabricated gold nanoparticles, allowing them to detect horseradish peroxidase at a concentration of 350 fM from 84 particles. The use of the highly optically dense DAB to enhance the LSPR shift is an interesting approach and is among other potential enzyme catalysed LSPR enhancement systems used [104,105]. The LSPR-enhancement by DAB has been further improved through the fabrication of a nanoarray based system and has allowed for the detection of variations caused by single enzymes and the analysis of the spectra of 700 Au nanorods, in an array, simultaneously [106]. The use of this nanoarray-based method, in conjunction with the advanced spectral acquisition technique, is advantageous as it allows for the extremely sensitive detection of biomolecules. Further development upon similar fabrications can potentially allow for highly multiplexed biosensing that retains sensitivity at a single particle level.

Thus far, the advancement of single particle spectroscopy methods have been shown to overcome the major challenges faced by nanoplasmonic biosensors. Taking these techniques and displaying detection schemes for the eventual application and commercialization of these biosensors has yet to be discussed. Through the modification of Au nanospheres and Au nanorods the detection of biomarkers in samples can now be explored and developed further. Modification of Au nanospheres with thiolated-DNA sequences is a commonly used method to create specificity to matched-DNA or RNA sequences [85,86,107]. Recently, such techniques have been adapted to work at a single particle level. The detection of human topoisomerase was demonstrated through the relaxation of super-coiled DNA and have reported potential detection limits similar to that of traditional SPR sensors [108]. Similarly, the detection of target miRNA and DNA was detected by monitoring single Au nanoparticles (75 nm), with a change in DNA confirmation, from hairpin to bound, resulting in the refractive index shift and allowed for a low limit of detection down to 3 nM [109]. Recently, Sim and co-workers [110] have developed a novel multiplexed sensing platform, with different sites for binding recognition, allowing for the detection of the cancer biomarkers α-fetoprotein, carcinoembryonic antigen and prostate specific antigen at 91, 94 and 10 fM respectively. Pushing the limit of detection to pico or even attomolar concentrations is one of the goals of biosensors and achieving that will potentially allow for early detection of diseases, such as cancer, which could lead to improved treatment efficacy [111,112].

**Figure 4:**
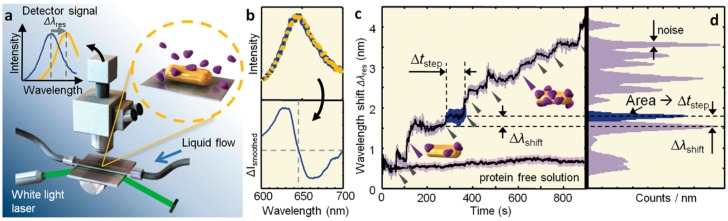
(**a**) The optical setup for the total internal reflection and illumination of a single gold nanorod (magnified) for the detection of single protein binding. Inset at the top left is the theoretical LSPR shift that is expected in response to a large increase in local refractive index, such as the binding of proteins. Top panel in (**b**) shows the spectrum of a single Au nanorod, before and after the binding of single proteins. The bottom panel in (**b**) shows the difference in intensity between pre and post-binding of the protein; (**c**) The resonance wavelength of the single Au nanorod, monitored as a function of time, shows multiple single protein binding events, while the resonance wavelength remains flat for the protein-free solution. The peaks in the histogram (**d**) correspond to single protein-binding events. Reprinted with permission from [70]. Copyright 2012 American Chemical Society.

**Figure 5:**
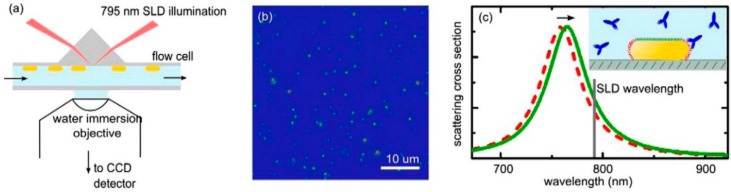
(**a**) Schematic diagram of the setup used to illuminate Au nanorods with a superluminescent diode through a glass prism. (**b**) Image of the modified Au nanorods on the surface obtained with the CCD and (**c**) illustration of the red-shift in LSPR scattering during the binding of proteins to the Au nanorod (inset). Reprinted with permission from [114]. Copyright 2015 American Chemical Society.

Single molecule sensitivity is considered an important goal for these types of sensors. Using photothermal microscopy, Zijlstra *et al.* have shown the real-time analysis of single protein binding events on a single gold nanorod with high sensitivity (see Figure 4) [67]. Similarly, another technique for detecting single protein binding events has also been developed, whereby an intense light source (white light laser) is used to illuminate the Au nanorods and is detected by an intensified CCD camera [113]. Using this method improves the signal to noise ratio and allows for the sensitive determination of LSPR shifts of single nanoparticles within milliseconds [113]. Both these approaches employ specialised equipment to achieve the desired level of sensitivity and are limited by the number of nanoparticles that can be monitored simultaneously. In order to achieve sufficient data about molecular properties of proteins, it is required to probe more particles at a single particle level. Recently, a new setup was developed based on prism-type total internal reflection microscopy, where Au nanorods were illuminated by a superluminescent diode, permitting the examination of hundreds of single particles for single protein binding events (see Figure 5) [114]. The development of this technique has enabled the monitoring of stochastic protein binding to Au nanorods, allowing us to probe for information about single protein binding.

The main limitation with single particle spectroscopy techniques seems to be obtaining information from a sufficient number of particles. With single particle spectroscopy techniques, which use a coupled spectrograph, large amounts of nanoparticles have been analysed. However, the amount of particle spectral information obtained is related to the time taken to scan across the surface. A different approach has recently been developed, where Au nanoparticles are imaged using DFM but the analysis is done through an algorithm processing of the red, green and blue (RGB) colour information of the particles [115]. They demonstrate the ability to image single Au nanospheres in intracellular environments and are able to quantify LSPR shifts, induced by NADH present in the cell. This is an interesting approach as it could potentially allow high-throughput studies for biosensing applications.

## 6. Conclusions with Opportunities and Future Direction

In summary, there have been significant advances in the design and characterization of single surface plasmon sensors. This is largely due to the advances in chemical synthesis of plasmonic nanostructures, the availability of new optical characterization techniques and numerical modelling tools that aid in the design of plasmonic sensors. As the potential benefits of this field are emerging, work on different synthetic strategies has led to the development of several protocols for the preparation of monodisperse plasmonic nanoparticles. There is also a vast literature on the purification and functionalization of plasmonic nanoparticles, thereby making it easy to fabricate plasmonic nanosensors. The different approaches taken for the detection of single molecules with plasmonic nanoparticles are encouraging.

The significant challenge in this area is finding ways to translate these approaches to point-of-care based clinical diagnostics. Plasmonic nanoparticles are currently employed in several clinical diagnostic tests for the detection of important biomarkers for cancer, HIV and other diseases [116]. Non-specific binding of other biomolecules plays an important role in the practical construction of these biosensors [117]. The formation of a protein-corona around metal nanoparticles when they are introduced to a cellular environment is well-documented [118]. The design of single nanoparticle sensors need to account for factors as non-specific binding arising from protein corona which might introduce spectral shifts. With careful adaption and further advances in the single nanoparticle-based sensing techniques, we believe that plasmonic nanoparticle based point-of-care sensing devices with single molecule sensitivity can realised. However, these methods do not exist in a vacuum, they compete with other fluorescence-based techniques that have single molecule sensitivity. Unlike fluorophores, plasmonic nanoparticles do not suffer from limitations, such as blinking or photobleaching, and can be employed to form label-free biosensors. There are significant challenges and opportunities in this domain. There is a need for new optical characterization and analysis approaches that can significantly increase the number of nanoparticles that can be probed simultaneously. The number of nanoparticles probed currently is small (hundreds to thousands), automation of optical microscope could be one solution. Multiplexing is another option, as several biomarkers can be detected at the same time. While, a large number of studies have focused on Au nanorods, multiplexing may be achieved by using plasmonic particles of different shape and composition.
